# Comparative proteomic analysis of cell lines and scrapings of the human intestinal epithelium

**DOI:** 10.1186/1471-2164-8-91

**Published:** 2007-04-03

**Authors:** Kaatje Lenaerts, Freek G Bouwman, Wouter H Lamers, Johan Renes, Edwin C Mariman

**Affiliations:** 1 Maastricht Proteomics Center, Nutrition and Toxicology Research Institute Maastricht (NUTRIM), Department of Human Biology, Maastricht University, PO Box 616, 6200 MD Maastricht, The Netherlands; 2 AMC Liver Center, Academic Medical Center, University of Amsterdam, Meibergdreef 69, 1105 BK Amsterdam, The Netherlands

## Abstract

**Background:**

*In vitro *models are indispensable study objects in the fields of cell and molecular biology, with advantages such as accessibility, homogeneity of the cell population, reproducibility, and growth rate. The Caco-2 cell line, originating from a colon carcinoma, is a widely used *in vitro *model for small intestinal epithelium. Cancer cells have an altered metabolism, making it difficult to infer their representativity for the tissue from which they are derived. This study was designed to compare the protein expression pattern of Caco-2 cells with the patterns of intestinal epithelial cells from human small and large intestine. HT-29 intestinal cells, Hep G2 liver cells and TE 671 muscle cells were included too, the latter two as negative controls.

**Results:**

Two-dimensional gel electrophoresis was performed on each tissue and cell line protein sample. Principal component and cluster analysis revealed that global expression of intestinal epithelial scrapings differed from that of intestinal epithelial cell lines. Since all cultured cell lines clustered together, this finding was ascribed to an adaptation of cells to culture conditions and their tumor origin, and responsible proteins were identified by mass spectrometry. When investigating the profiles of Caco-2 cells and small intestinal cells in detail, a considerable overlap was observed.

**Conclusion:**

Numerous proteins showed a similar expression in Caco-2 cells, HT-29 cells, and both the intestinal scrapings, of which some appear to be characteristic to human intestinal epithelium *in vivo*. In addition, several biologically significant proteins are expressed at comparable levels in Caco-2 cells and small intestinal scrapings, indicating the usability of this *in vitro *model. Caco-2 cells, however, appear to over-express as well as under-express certain proteins, which needs to be considered by scientists using this cell line. Hence, care should be taken to prevent misinterpretation of *in vitro *obtained findings when translating them to the *in vivo *situation.

## Background

*In vitro *cell models play an important role in understanding cellular events related to (patho)physiological conditions in humans. Many advantages such as accessibility, homogeneity of the cell population, reproducibility, growth rate, and hence the amount of material for analysis make them indispensable study objects in the search for molecular mechanisms. The differentiation process of intestinal cells, pathologies related with inflammatory conditions in the intestine, but also the adaptation of intestinal cells to certain nutritional conditions are often studied by an *in vitro *approach using intestinal epithelial cell models [1-5]. The human Caco-2 and HT-29 cell lines are commonly used models representing features of the human intestinal epithelium. Caco-2 cells were derived from a human colon adenocarcinoma, and they differentiate spontaneously *in vitro *under standard culture conditions thereby exhibiting enterocyte-like structural and functional characteristics [6]. In differentiated state, they mimic typical characteristics of the human small intestinal epithelium, like a well-developed brush border with associated enzymes such as alkaline phosphatase and sucrase isomaltase [6]. Nevertheless, the Caco-2 cell model is different from the small intestine in several aspects, and their phenotype is dependent on the time in culture [7,8]. The HT-29 cell line is also of human colon adenocarcinoma origin, and cannot differentiate spontaneously *in vitro *under standard conditions, representing undifferentiated colonic epithelial cells [9].

Caco-2, HT-29 cells, and many other widely used cell lines originate from tumour tissue, which alters the cellular metabolism drastically compared to physiological conditions. Therefore, it is difficult to interpret how representative a specific cell line is for the tissue from which it is derived. Furthermore, in comparison with the *in vivo *situation with influences from different neighbouring cells, (neuro)endocrine regulators and blood flow, the *in vitro *model is relatively simple. Although this is ideal for specific research questions, it complicates the translatability of *in vitro *results to *in vivo *situations. Above all, it is not clear if cells in culture retain the gene expression profiles of their *in vivo *counterparts. A study regarding the gene expression patterns in 60 human cancer cell lines, including colon cancer cell lines, revealed that the tissue from which the cells are derived is the main factor accounting for the variation in gene expression [10]. Gene expression was also investigated at a large scale in Caco-2 and HT-29 cells using microarray technology [11]. Another study revealed novel proteins which were associated with proliferation and differentiation in Caco-2 cells in relation to colon carcinogenesis [12]. However, differentiated Caco-2 cells, and also HT-29 cells are often used to reproduce features of the intestinal epithelium *per se*. 

A suitable and innovative method to evaluate the validity of such models for the 'normal' epithelium is by investigating similarities and differences in protein expression between cultured intestinal epithelial cells and epithelial scrapings obtained from human intestine. Protein isolates of cultured Caco-2 cells and HT-29 were subjected to two-dimensional gel electrophoresis (2-DE) and compared with the 2-DE patterns generated from normal epithelium from the small and large intestine. The Caco-2 cells are very often cultured on permeable supports. This way of culturing polarized cells resembles the *in vivo *situation more closely, as the cells can take up and secrete molecules on both sides of the monolayer. The supports that were applied in this study were collagen-coated, as this has been shown to promote cell attachment and growth leading to confluence of the Caco-2 monolayer more rapidly [13]. In addition, ultrastructural features of the cell layer were thoroughly investigated in that study and appeared to mimic those of the small intestinal epithelium, which is relevant for a good cell model. As a negative control, protein profiles of the human Hep G2 hepatoma cell line (liver) and the TE 671 rhabdomyosarcoma cell line (muscle) were used.

## Results

### Protein expression profiles of the intestinal scrapings and cell lines

**Figure 1:**
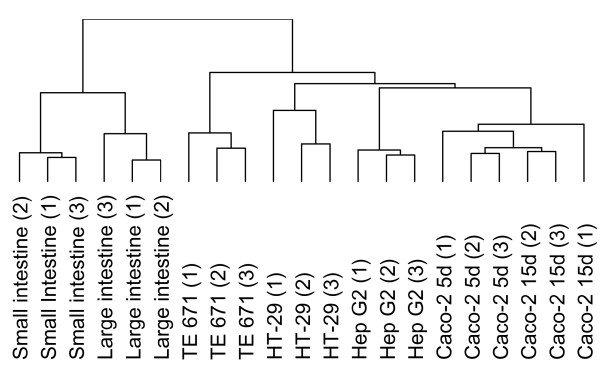
**Unsupervised hierarchical clustering of protein profiles**. A dendogram, generated by unsupervised hierarchical clustering of protein expression data of 2-D profiles of protein isolates obtained from three biological replicates of small intestinal scrapings, partially and fully differentiated Caco-2 cells, HT-29 cells, Hep G2 cells and TE 671 cells. Profiles of large intestine (1) and (2) come from one human sample.

For each tissue or cell line under study, three replicate gels were made of biological independent samples, except for the large intestinal scrapings, of which only 2 independent samples could be obtained. A dendogram (Figure 1) generated by unsupervised hierarchical clustering of the expression data of 2-D patterns revealed that the profiles of the epithelial scrapings of both small and large intestine were separated from those of the cell lines. In the cell line cluster, profiles of cells of epithelial origin (Caco-2, HT-29 and Hep G2) were separated from the profile of cells of mesenchymal origin (TE 671). Differentiated as well as undifferentiated TE 671 cells were tested, but the 2-D patterns of these cells were similar. The differentiation process induced some obvious protein changes, such as an up-regulation of expression of the spot which contains vimentin. However, overall expression of the undifferentiated and differentiated TE 671 cells were alike and clustered together in all analyses described below. Therefore, only data of the differentiated cells were shown in detail, as the main focus is the behaviour of the intestinal cells.

**Figure 2:**
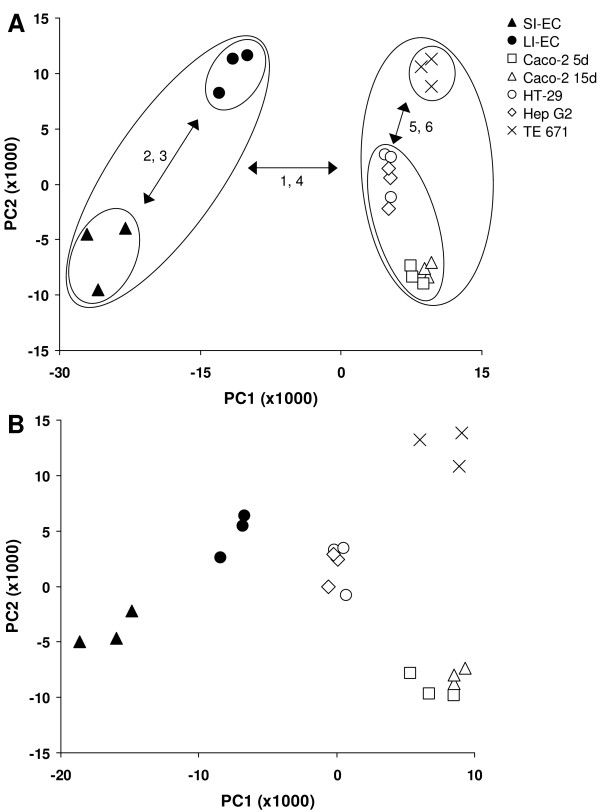
**PCA of protein profiles**. PCA projection of protein profiles obtained by 2-DE in a scatter plot using the entire data set (A) and the data set after subtracting data points corresponding to proteins common for *in vitro *cell cultures (B). In panel A, PC1 and PC2 explain 37.3% and 13.8% of the total variation, respectively. In panel B, PC1 and PC2 explain 24.9% and 17.4% of the total variation, respectively. Open symbols and closed symbols represent 2-D profiles of cell lines and epithelial scrapings, respectively, as shown in panel A. The cluster numbers containing proteins which are mainly responsible for separation between groups are depicted next to the arrows in panel A. SI, small intestine; LI, large intestine; EC, epithelial cells.

To get further insight in how 2-D patterns of intestinal Caco-2 cells (partially and fully differentiated) and HT-29 cells are related to profiles of small and large intestinal epithelial scrapings, a principal component analysis (PCA) was performed. PCA allows for grouping of cells with overall similar protein expression characteristics and for identifying proteins which are responsible for the differences between the groups. The plot shown in Figure 2A is a simple scatter plot of the first two principal components (PC). PC1 and PC2 explain 37.3% and 13.8% of the total variation, respectively. Triplicate gels cluster together which indicates that the biological variation is responsible for the clear separation of the different samples. Along PC1, three distinct groups are visible, of which one corresponds to all cultured cell lines, independent of tissue origin. In the cluster of the cell lines, three sub-clusters exist along PC2 corresponding to profiles from TE 671 cells, HT-29 together with Hep G2 cells and Caco-2 cells, independent of their stage of differentiation. When blood-derived proteins are removed from the data set, the PCA plot is very similar but PC1 explains only 29.8% of the total variation, largely because profiles of the small and large intestinal cells move closer towards the cell lines along PC1, whereas PC2 explains 15.7% (data not shown). In addition, removing the blood proteins for generating the dendogram did not influence the branching.

Protein data of all *in vitro *cell cultures were used to generate a set of protein spots (within a circle of 25% from the plot centre), which have the same relative abundance in all types of cell lines studied. These proteins are present in all *in vitro *cultured cells included in this analysis, and seem not much contributory to the specific phenotype of a certain cell line. Therefore, another PCA was performed, in which expression data of the 25% proteins common for *in vitro *cell cultures were subtracted from the data set (without blood proteins), so proteins that make a cell line unique contribute more to the overall pattern. The outcome of the analysis, displayed as a scatter plot of PC1 and PC2, revealed that protein expression patterns of scrapings and cell lines are still separated (Figure 2B). Along PC1 (explaining 24.9% of the variation), the 2-D profiles of the HT-29 cells move in the direction of the profiles of the colonic scrapings. Along PC2 (explaining 17.4% of the variation) the profiles of Caco-2 cells appear at a similar position as those of the small intestinal cells, but this was already visible in Figure 2A. Along PC1 and PC2, the profiles of the TE 671 cells move further away from the profiles of all other, epithelial cells.

### Protein expression in the intestinal scrapings and cell lines

**Figure 3:**
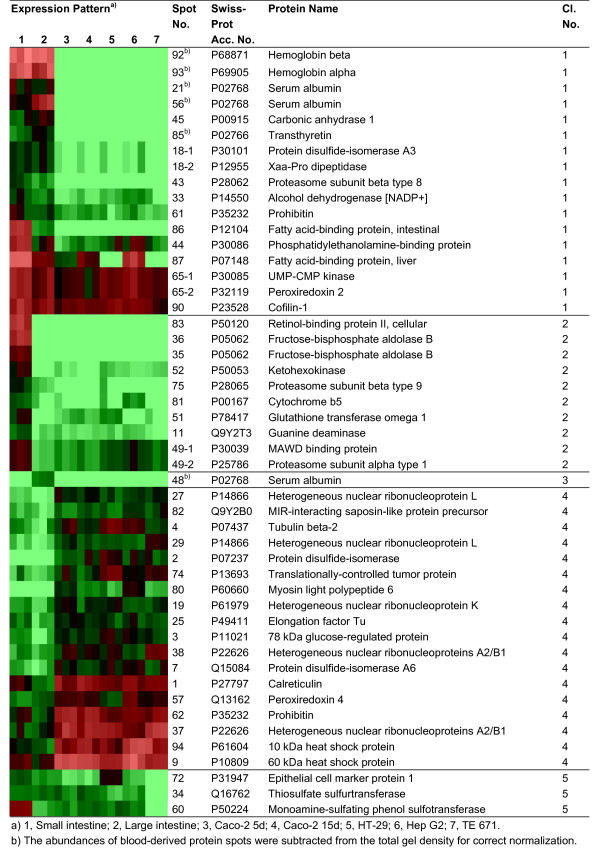
**Clustering of identified protein spots according to the expression levels in analyzed samples**. Identified protein spots grouped in different clusters (cluster 1–5) according to the expression level in different samples. Triplicate spot intensities are shown by a colour range; bright red, black, and bright green represent high, average, and low levels of protein expression.

**Figure 4:**
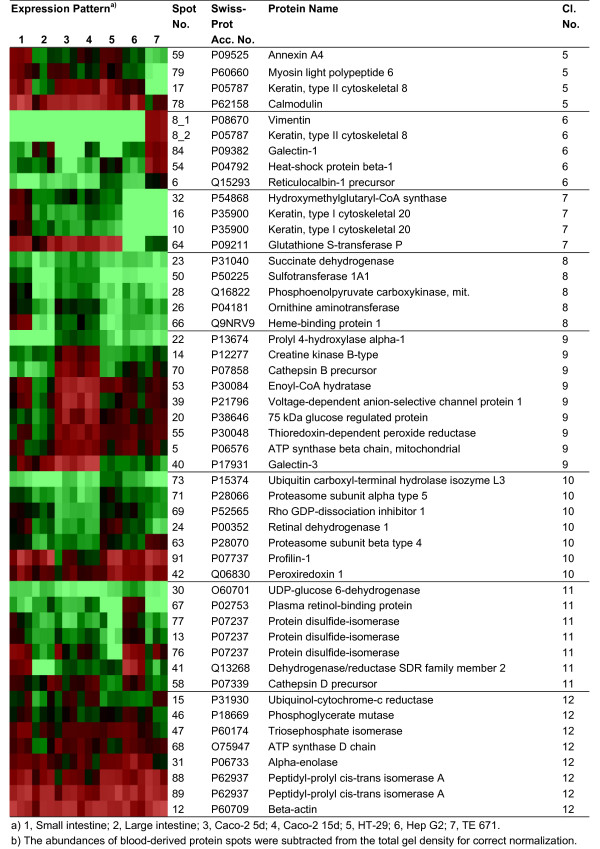
**Clustering of identified protein spots according to the expression levels in analyzed samples (continuation of Figure 3)**. Identified protein spots grouped in different clusters (cluster 5–12) according to the expression level in different samples. Triplicate spot intensities are shown by a colour range; bright red, black, and bright green represent high, average, and low levels of protein expression.

**Figure 5:**
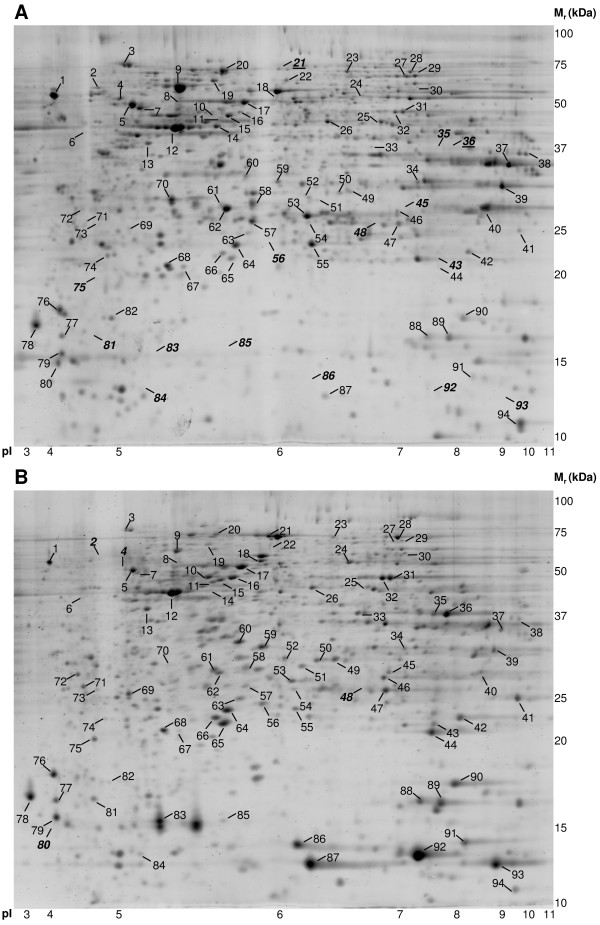
**Protein profiles of differentiated Caco-2 cells and human small intestinal epithelial cells**. 2-D pattern of 5-day-differentiated Caco-2 cells (A) and small intestinal epithelial scrapings (B). Identified protein spots are indicated with spot numbers corresponding to those in Figure 3 and 4. When a spot number is indicated in bold/italic, this means that the spot could not be observed in the particular pattern.

**Figure 6:**
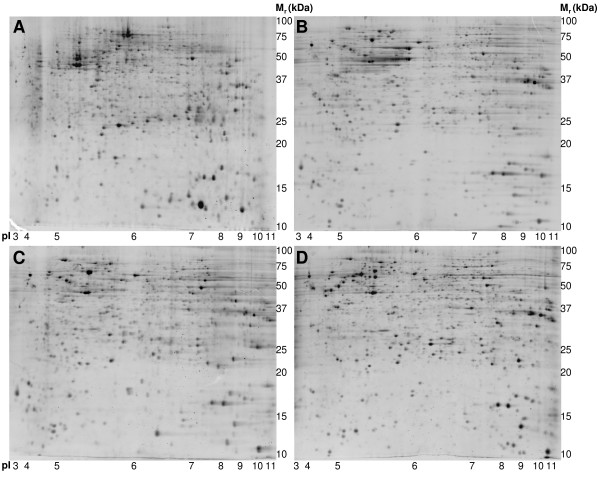
**Protein profiles of human large intestinal epithelial cells, HT-29, Hep G2, and TE 671 cells**. 2-D pattern of large intestinal epithelial scrapings (A), HT-29 cells (B), Hep G2 cells (C) and TE 671 cells (D).

In PDQuest, 2-D patterns of all protein isolates under study were compared and resulted in 131 differentially expressed proteins. In addition, 8 protein spots were selected that showed a similar expression pattern in all isolates. Out of 139 protein spots, 94 spots (68%) were identified by matrix-assisted laser desorption/ionization time-of-flight mass spectrometry (MALDI-TOF MS) [see Additional file 1 for identification characteristics]. Identified proteins were grouped in 12 clusters according to their expression pattern in the analyzed samples. These clusters of protein spots are shown in Figure 3 and 4, in which log-transformed expression values of the biological replicates of different samples are shown in color [See additional file 2 for the averaged spot intensities and the standard deviation of these spots in each tissue and cell line]. The spot numbers correspond to those indicated in Figure 5. In this figure, a 2-D pattern of 5-day-differentiated Caco-2 cells (Figure 5A), and a 2-D pattern of a small intestinal epithelial scraping (Figure 5B) are shown to give an indication of the overlap of the profiles. The spot numbers of spots which were not present in one of these patterns are indicated in bold, italic. Figure 6 shows 2-D patterns of large intestinal epithelium, HT-29, Hep G2 and TE 671 cells to obtain a general view on the differences and similarities of the protein expression.

Cluster 1 contains proteins which show a high expression in both intestinal epithelial scrapings relative to the *in vitro *cell cultures. Cluster 2 and 3 consist of proteins with a high expression level in small and large intestinal scrapings, respectively. Proteins with low expression levels in the scrapings are grouped in cluster 4. Cluster 5 contains proteins with a high expression in all cells of epithelial origin, and cluster 6 contains proteins with a low expression in those cells. Cluster 7 shows proteins with a high expression in all protein isolates of intestinal origin, and protein spots with a high expression in small intestinal epithelium and Caco-2 cells are shown in cluster 8. Cluster 9 and 10 represent proteins with a high and low expression in Caco-2 cells, respectively. Cluster 11 contains proteins mainly expressed in Hep G2 but with co-expression in other relevant cell types. The last cluster, cluster 12 shows proteins with a relatively similar expression level in all protein isolates.

## Discussion

The current proteomics approach aims to obtain a better insight in the expression patterns of human intestinal epithelial cells and the commonly used *in vitro *models for these cells, Caco-2 cells and HT-29 cells, both derived of human intestinal adenocarcinoma. This information is highly relevant to researchers using such models, and aiming for translating data to the *in vivo *situation.

When comparing the 2-D pattern of Caco-2 cells and small intestinal cells, it becomes apparent that many protein spots are present in both profiles, indicating a great deal of overlap in the type of proteins expressed, supporting the usability of this *in vitro *model. However, several high abundant proteins in human epithelial cells of the small intestine are not expressed or expressed at low levels in Caco-2 cells, and *vice versa*. This might be caused by the transformed status of Caco-2 cells and an adaptation of their metabolism to growth conditions outside the body. This is supported by the clustering data, which revealed a separation of the epithelial scrapings and all *in vitro *cell lines, irrespective of their tissue origin, suggesting that the protein expression profiles were quite similar among each of the cell lines, but different from the protein profiles of epithelium *in vivo*. When exploring the clusters explaining the main variation in expression, noticeably, typical proteins with a high expression in the scrapings are hemoglobin alpha and beta, serum albumin and transthyretin, which appear to originate from blood cells. This finding demonstrates the difficulty of obtaining pure cell isolates, and points to the advantage of using *in vitro *cell lines, which consist of a homogenous cell population.

Several other proteins are responsible for the distinction of the *in vitro *and *in vivo *proteomes. First, intestinal fatty-acid binding protein (FABP) expression is only observed in the protein profiles of intestinal scrapings, and the expression is highest in small intestinal epithelium. This was observed in another study as well, in which levels were tested at different segments of the human intestinal tract [14]. Liver FABP is highly expressed in intestinal scrapings, but shows expression in liver Hep G2 and Caco-2 cells too. In the latter cells, its expression increases with differentiation, which is in agreement with a previous study [15]. Another member of the FABP family is cellular retinol-binding protein (RBP) II, which is solely expressed in the small intestinal epithelial scrapings. This vitamin A-binding protein is abundant in the human small intestinal mucosa and is localized to the villus-associated enterocytes by immunohistochemistry [16]. In adult rats, expression of cellular RBP II is restricted to villus-associated enterocytes in the proximal small intestine [17]. This protein may be important for intestinal absorption and/or metabolism of retinol. It is also reported in literature that Caco-2 cells express the protein, and that the expression increases with differentiation [18], but we failed to detect the protein in the profiles of these cells. Two proteins involved in fructose metabolism, ketohexokinase and fructose-bisphosphate aldolase B, show a high expression in the small intestine. The latter enzyme is known to be primarily expressed in liver, small intestine, and kidney [19]. In Hep G2 cells, aldolase B mRNA was shown to be weakly expressed [20]. In comparison, differentiated Caco-2 cells express fructose-bisphosphate aldolase A and C mRNA [21], and recently, we identified isoform A protein in Caco-2 cells [22]. To recapitulate, several proteins with a role in lipid and carbohydrate metabolism are higher expressed, or exclusively expressed in the *in vivo *intestinal material. This may be a reflection of the intestine's capacity in absorbing and metabolizing complex mixtures of nutrients, whereas *in vitro *cells are probably adapted to the culture medium with a rather simple composition.

Prohibitin is present as two spots in the gels, from which one spot (no. 61, cluster 1) shows a strong expression whereas the other spot (no. 62, cluster 4) shows a low expression in the epithelial scrapings, compared to the cultured cells. Similarly, two spots were identified as myosin light polypeptide 6 (no. 80, cluster 4 and no. 79, cluster 5) from which one spot is absent in the epithelium *in vivo*, while the other spot is as abundant as in most cell lines. These data indicate that gene products might be processed via alternative pathways in *in vitro *cell cultures. Next to specific forms of prohibitin and myosin light polypeptide 6, we identified more proteins with a typically higher expression in all cultured cells tested, and they account also for the separation of *in vitro *and *in vivo *proteomes. The higher expression levels of proteins discussed below might be explained by the tumoural origin of the *in vitro *cell lines tested here. Heterogeneous nuclear ribonucleoproteins (HnRNPs) K, L and A2/B1 belong to a family of proteins with central roles in DNA repair, telomere elongation, cell signalling and in regulating gene expression at transcriptional and translational level. Through these key cellular functions, individual hnRNPs have a variety of potential roles in tumour development, and several hnRNPs, under which hnRNP K and A2/B1 are up-regulated in various cancers [23]. Translationally-controlled tumour protein is also higher expressed in *in vitro *cultured cells. It is not a tumour-specific protein, although its expression level tends to be higher in tumours, compared to the corresponding normal tissue [24]. The protein is highly conserved and widely expressed in all eukaryotic organisms, and is thought to be important for cell growth and division [25]. Several heat shock proteins (HSPs), such as 78-kDa glucose-regulated protein, 10-kDa HSP and 60-kDa HSP are also higher expressed in the cell lines. It was demonstrated before that HSPs, under which 78-kDa glucose-regulated protein, are expressed in increased amounts in many tumours [26]. Recently, 10-kDa HSP was shown to be increased in colorectal cancer tissue compared to normal tissue [27]. Elevated HSP expression has a cytoprotective role. For example, individual HSPs are able to block the pathways of apoptosis, and hence appear to play a role in tumour pathogenesis.

A set of proteins was higher expressed in all cells of epithelial origin included in this analysis. Two enzymes with a role in detoxification, monoamine-sulphating phenol sulfotransferase and thiosulfate sulfurtransferase, show such an expression pattern. Expression of monoamine-sulphating phenol sulfotransferase was already demonstrated in the human jejunal mucosa and the Caco-2 cell line [28,29]. Thiosulfate sulfurtransferase activity was present in the small intestine, colon and rectum of human [30], but high activity of this enzyme was also shown in liver and kidney of rat [31]. Annexin A4 shows a comparable expression pattern. The protein belongs to a ubiquitous family of Ca2+-dependent membrane-binding proteins thought to be involved in membrane trafficking and membrane organization within cells and is found at high levels in many epithelial cells, where it is closely associated with the apical region [32]. Epithelial cell marker protein 1, also called stratifin or 14-3-3 sigma, was also highly expressed in the epithelial cells but was not observed in the mesenchymal TE 671 cells. These data indicate that Caco-2 cells as well as HT-29 cells express proteins that are characteristic to human intestinal epithelium *in vivo*, which is further supported by the findings mentioned in next paragraph.

Proteins with a higher expression in the intestinal cell lines Caco-2 and HT-29, and in both the small intestine and colon epithelial scrapings, compared to negative controls Hep G2 and TE 671, are hydroxymethylglutaryl-CoA synthase, keratin type 1 cytoskeletal 20, and glutathione S-transferase P. These proteins could have an important role in gut epithelial cells, as all profiles of intestinal origin, being from *in vitro *cultures or scrapings, express them. Keratin type 1 cytoskeletal 20 is such a protein identified as a major cytoskeletal polypeptide of the human intestinal epithelium [33], and is thought to have a role in the intermediate filament organization in intestinal epithelium [34]. Hydroxymethylglutaryl-CoA synthase is a control enzyme in the synthesis of ketone bodies, and is highly expressed in liver and colon, and low in testis, heart, skeletal muscle and kidney [35]. Expression in the human small intestine is reported here for the first time.

Next, Caco-2 cells share common characteristics with small epithelial scrapings. Proteins with a similar expression in both are succinate dehydrogenase, sulfotransferase 1A1, mitochondrial phosphoenolpyruvate carboxykinase, ornithine aminotransferase, and heme-binding protein 1. They can be used as markers for the small intestinal epithelial phenotype of Caco-2 cells. Ornithine aminotransferase appears to be an important protein in the intestine, and is down-regulated by glutamine depletion in Caco-2 cells and starvation in mouse small intestine [4,36]. This key enzyme is present predominantly in the small intestine and is involved in the conversion of glutamine to ornithine. Ornithine is a precursor for polyamines, known for their involvement in cell proliferation, cell differentiation and repair for intestinal cells [37]. Mitochondrial phosphoenolpyruvate carboxykinase has an important role in gluconeogenesis, where it catalyzes the conversion of oxaloacetate to phosphoenolpyruvate. We found that glutamine depletion in Caco-2 cells resulted in a decreased expression of this protein [4]. From above data, one can deduce that the Caco-2 cells express biologically important proteins at a similar level as in the small intestine. Such typical similarities in expression are not observed between Caco-2 cells and large intestinal scrapings.

Proteins with a higher or lower expression in Caco-2 cells, compared to all other protein isolates, were also observed, and this information needs to be considered when studying processes in which one of these proteins play an important role. Mostly, protein expression of partially differentiated Caco-2 cells equals that of fully differentiated Caco-2 cells. Hep G2 cells have the highest plasma RBP levels, which is in line with the liver being the major site for plasma RBP synthesis, where it is secreted in the blood for retinol transport to the peripheral tissues. However, other sites of plasma RBP secretion are reported [38]. Epithelial scrapings of the small and the large intestine contain this protein as well; however, we cannot exclude the possibility that it is derived from blood. The finding that plasma RBP is expressed at comparable levels in Caco-2 cells supports the idea that intestinal cells can synthesize the protein too. Recently, we have shown that plasma RBP was down-regulated considerably by glutamine deficiency in Caco-2 cells [4], and this may have relevance for the intestinal condition *in vivo*. Proteins with a role in cytoskeletal function and proteasome activity are present throughout different clusters, which makes it difficult to interpret data concerning these central cellular processes. Some proteins involved in carbohydrate metabolism, namely triosephosphate isomerase, phosphoglycerate mutase and alpha enolase are similarly expressed in all samples (cell lines and scrapings) tested.

## Conclusion

This study was designed to compare expression profiles of widely used intestinal cell models with their *in vivo *counterparts. PCA revealed that global protein expression of intestinal epithelial scrapings differed considerably with that of intestinal epithelial cell lines. In the cell lines, explicit, highly expressed proteins probably correspond to their tumoural origin, whereas several proteins with a low or undetectable expression could be explained by their adaptation to culture conditions. Numerous proteins showed a similar expression in Caco-2 cells, HT-29 cells, and both the intestinal scrapings, of which some appear to be characteristic to human intestinal epithelium *in vivo*. In addition, several biologically significant proteins are expressed at comparable levels in Caco-2 cells and small intestinal scrapings, indicating the usability of this *in vitro *model. Caco-2 cells, however, appear to over-express as well as under-express certain proteins, which needs to be considered by scientists using this cell line. As a result, care should be taken to prevent misinterpretation of *in vitro *obtained findings when translating them to the *in vivo *situation.

## Methods

### Materials

The Caco-2 and Hep G2 cell lines were obtained from the American Type Culture Collection (Rockville, MD, USA). The HT-29 and TE 671 cell lines were kindly provided by Dr. J. Keijer (RIKILT, Wageningen, The Netherlands) and Dr. M. De Baets (Dept. Neurology, Academical Hospital Maastricht, Maastricht, The Netherlands), respectively. Dulbecco's modified Eagle's medium (DMEM) and cell culture supplements were purchased from Invitrogen (Carlsbad, CA, USA) except fetal calf serum (FCS) which was from Bodinco (Alkmaar, The Netherlands). CHAPS, dithiothreitol (DTT), iodoacetamide, α-cyano-4-hydroxycinnamic acid, alcohol dehydrogenase and adrenocorticotropic hormone fragment 18–39 were from Sigma (St. Louis, MO, USA). Urea, SYPRO Ruby Protein Stain and all reagents for SDS-PAGE were from Bio-Rad Laboratories (Hercules, CA, USA). Immobiline Dry Strips (pH 3–11, nonlinear) and immobilized pH gradient (IPG) buffer (pH 3–10, nonlinear) were from Amersham Biosciences (Little Chalfont, England). Sequencing grade modified trypsin was obtained from Promega (Madison, WI, USA).

### Cell culture

All cells were cultured in DMEM supplemented with 1% v/v non-essential amino acid solution, 100 units/mL penicillin and 100 μg/mL streptomycin. For the Caco-2 cell line, 20% v/v FCS was added to this medium, whereas 10% v/v FCS was added for the other cell lines. Caco-2 cells were grown until confluence for 6 days onto 24 mm Transwell bicameral systems (Corning, Aston, MA, USA) with collagen-coated membranes (0.4 μm pore size, 4.7 cm2 surface area). After this period, cells were maintained in culture to differentiate for 5 and 15 days, representing a partially and a fully differentiated enterocytic phenotype, respectively. Human Hep G2 and HT-29 cells were cultured until confluence. The human muscle cell line TE 671 underwent myogenic differentiation for five days with 10^-7 ^M 12-O-tetradecanoylphorbol-13-acetate (TPA) [39,40]. We also tested TE 671 cells not differentiated with TPA, but 2-D patterns were similar and for that reason left out of all analyses.

### Sample preparation

Cell lines: the cells were washed three times with PBS. Proteins were isolated from the cell lines by scraping them in ice-cold PBS, and centrifuging the obtained cell suspensions at 350 g for 5 min at 4°C. Cell pellets were dissolved in a cell lysis buffer containing 8 M urea, 2% (w/v) CHAPS, 65 mM DTT, 0.5% (v/v) IPG buffer supplemented with Complete protease inhibitors (Roche Applied Science, Indianapolis, IN, USA). This mixture was subjected to three cycles of freeze thawing, vortexed thoroughly and centrifuged at 20000 g for 30 min at 10°C. Supernatant was collected and stored at -80°C until further analysis. For each cell line, protein isolates were obtained from three independent cultures.

Epithelial scrapings: Epithelial scrapings of three small intestinal samples and two colon (sigmoid colon) samples were obtained from patients who underwent Whipple pancreatico-duodenectomy and colectomy as a treatment for pancreas and colon cancer, respectively. Such surgery implicates that large sections of the patients' intestine need to be removed. The epithelium was scraped from that intestinal tissue at least 10 cm removed from the tumour. The samples were snap frozen in liquid nitrogen and stored at -80°C until proteins were isolated. For protein isolation, samples were grinded in liquid nitrogen, after which cell lysis buffer (see above) was added. The rest was performed as described for the cell line material. The samples were not pooled, as enough protein material could be obtained by the scraping. An informed consent was obtained from the patients.

The protein concentrations of all protein samples were determined by a Bradford based protein assay [41].

### 2-DE

The 2-DE procedure was performed as described with minor modifications [4]. Briefly, 200 μg of total protein was separated by isoelectric focusing using Immobiline Dry Strips (24 cm, pH 3–11, nonlinear) according to the following protocol: 12 h at 30 V, 1 h at 500 V, 1 h at 1000 V, 2 h gradient from 1000 to 8000 V, 35 kVh at 8000 V. Strips were equilibrated and placed onto 12.5% SDS-polyacrylamide gels for protein separation in the second dimension. Electrophoresis was conducted at 200 V constantly for 6 h in a 24 cm Protein Dodeca Cell (Bio-Rad Laboratories). The gels were stained with SYPRO Ruby Protein Stain according to the manufacturer's protocol and the proteins were visualized by scanning gels with the Molecular Imager FX (Bio-Rad Laboratories).

### Gel image and data analysis

Spot detection, matching and the examination of differentially expressed proteins was performed using PDQuest v7.3 (Bio-Rad Laboratories). Three biological replicates were made per condition and formed one replicate group with average normalized spot intensities. Initially, spot abundances were normalized with respect to the total gel density. However, to evaluate the effect of the contaminating, blood-derived proteins, the abundances (see Additional file 2) were normalized with respect to the total gel density subtracted with the density of blood proteins. A spot was regarded as significantly differentially expressed between groups if the average spot intensity differed 1.4-fold or more and if *P *< 0.05 (Student's t-test) for at least one comparison. GeneMaths XT software (Sint-Martens-Latem, Belgium) was used to produce a dendogram using the unsupervised hierarchical clustering method Unweighted Pair Group Method with Arithmetic Means (UPGMA) based on the Pearson correlation coefficients. In addition, PCA was applied on the protein data, and both were used as exploratory data analysis tools to compare protein spot patterns derived from the used tissues and cell lines.

### In-gel digestion

For identification, protein spots were excised from the gel using an automated spot cutter (Bio-Rad Laboratories) and processed on a MassPREP digestion robot (Waters, Manchester, UK). A solution of 50 mM ammonium bicarbonate in 50% v/v acetonitrile was used for SYPRO Ruby destaining. Cysteines were reduced with 10 mM DTT in 100 mM ammonium bicarbonate for 30 minutes followed by alkylation with 55 mM iodoacetamide in 100 mM ammonium bicarbonate for 20 minutes. Spots were washed with 100 mM ammonium bicarbonate to remove excess reagents and were subsequently dehydrated with 100% acetonitrile. Trypsin (6 ng/μl) in 50 mM ammonium bicarbonate was added to the gel plug and incubation was preformed at 37°C for 5 h. The peptides were extracted with 1% (v/v) formic acid/2% (v/v) acetonitrile.

### MALDI-TOF MS

For MALDI-TOF MS 1.5 μl of each peptide mixture and 0.5 μl matrix solution (2.5 mg/ml α-cyano-4-hydroxycinnamic acid in 50% acetonitrile/0.1% TFA) was spotted automatically onto a 96 well-format target plate. The spots were allowed to air dry for homogeneous crystallization. Spectra were obtained using an M@LDI-LR mass spectrometer (Waters). The instrument was operated in positive reflector mode. Acquisition mass range was 800–4000 Da. The instrument was calibrated on 10–12 reference masses from a tryptic digest of alcohol dehydrogenase. In addition, a near point lockmass correction for each sample spot was performed using adrenocorticotropic hormone fragment 18–39 (MH+2465.199) to achieve maximum mass accuracy. Typically 120 shots were combined and background subtracted. A peptide mass list was generated for the subsequent database search [42,43].

### Database search

The peptide mass list generated with MassLynx v4.0.5 was searched with ProteinLynx Global Server v2.0.5 (Waters) or Mascot Search Engine [44] against the Swiss-Prot Database [45] for protein identification. Taxonomy was set to Homo sapiens and Mascot probability scores were calculated using 30 mass peaks or less with highest signal intensity, trypsin and keratin peaks were excluded. One miss-cleavage was tolerated; carbamidomethylation was set as a fixed modification and oxidation of methionine as an optional modification. The peptide mass tolerance was set to 100 ppm. No restrictions were made on the protein molecular weight and the isoelectric point. A protein was regarded identified when it had a significant Mascot probability score (scores greater than 60 correspond to *P *< 0.05), and at least four matched peptides, from which different forms of the same peptide were excluded.

## Authors' contributions

KL carried out cell culture, sample preparation of cell line and tissue material, contributed to the analysis and interpretation of data, and wrote the manuscript. FGB carried out the 2-DE and MALDI-TOF MS, analyzed the data and participated in writing the methods section of the manuscript. WHL collected the patient material. JR and ECM supervised the project and edited the manuscript. All authors read and approved the final manuscript.

## Supplementary Material

Additional file 1 Characteristics of protein identifications. Detailed protein identification characteristics of spot numbers indicated in Figure 5. Click here for file

Additional file 2 Spot intensities of identified proteins. Averaged spot intensities with standard deviations of identified protein spots which are displayed in Figure 3 and 4. Click here for file
