# Supplementary material for: Comparative proteomic analysis of cell lines and scrapings of the human intestinal epithelium
Source: BMC Genomics. 2007 Apr 3;8:91. doi: 10.1186/1471-2164-8-91 (PMC1852558; doi:10.1186/1471-2164-8-91)
Supplement: Additional file 1 — Characteristics of protein identifications. Detailed protein identification characteristics of spot numbers indicated in Figure 5. [file 1471-2164-8-91-S1.pdf]

Additional file 1 - Detailed protein characteristics of spot numbers indicated in Figure 5.

Spot No.	Swiss-Prot Acc. No.	Protein Name	M_r (kDa) th.	p*I* th.	MASCOT Score	Seq. Cov. (%)	Nr. of Pept. Searched	Nr. of Pept. Matched	Nr. of Pept. Unmatched
1	P27797	Calreticulin	48	4.29	153	44	27	11	11
2	P07237	Protein disulfide-isomerase	57	4.76	174	25	21	12	7
3	P11021	78 kDa glucose-regulated protein	72	5.07	233	38	44	19	20
4	P07437	Tubulin beta-2	50	4.78	180	32	34	12	16
5	P06576	ATP synthase beta chain, mitochondrial	57	5.26	237	46	30	17	7
6	Q15293	Reticulocalbin-1 precursor	39	4.86	82	30	42	8	32
7	Q15084	Protein disulfide-isomerase A6	48	4.95	148	28	13	9	3
8-1	P08670	Vimentin	54	5.06	76	16	23	8	14
8-2	P05787	Keratin, type II cytoskeletal 8	54	5.52	71	19	23	9	14
9	P10809	60 kDa heat shock protein	61	5.7	171	46	41	14	22
10	P35900	Keratin, type I cytoskeletal 20	48	5.52	206	48	38	17	17
11	Q9Y2T3	Guanine deaminase	51	5.44	96	35	53	13	40
12	P60709	Beta-actin	42	5.29	106	32	19	9	10
13	P07237	Protein disulfide-isomerase	57	4.76	157	26	25	13	11
14	P12277	Creatine kinase B-type	43	5.34	173	51	50	14	33
15	P31930	Ubiquinol-cytochrome-c reductase	53	5.94	69	14	26	7	18
16	P35900	Keratin, type I cytoskeletal 20	48	5.52	207	47	38	16	17
17	P05787	Keratin, type II cytoskeletal 8	54	5.52	125	39	47	15	26
18-1	P30101	Protein disulfide-isomerase A3	57	5.98	112	34	58	14	40
18-2	P12955	Xaa-Pro dipeptidase	54	5.64	83	27	58	11	45
19	P61978	Heterogeneous nuclear ribonucleoprotein K	51	5.39	72	18	17	6	10
20	P38646	75 kDa glucose regulated protein	74	5.87	153	28	22	14	7
21	P02768	Serum albumin	69	5.92	196	29	27	18	9
22	P13674	Prolyl 4-hydroxylase alpha-1	61	5.7	72	19	28	8	19
23	P31040	Succinate dehydrogenase	73	7.06	74	21	32	10	22
24	P00352	Retinal dehydrogenase 1	55	6.29	112	27	34	11	21
25	P49411	Elongation factor Tu	50	7.26	97	23	20	7	11
26	P04181	Ornithine aminotransferase	49	6.57	112	24	16	8	7
27	P14866	Heterogeneous nuclear ribonucleoprotein L	60	6.65	78	18	26	9	17
28	Q16822	Phosphoenolpyruvate carboxykinase, mit.	71	7.56	132	24	35	13	11
29	P14866	Heterogeneous nuclear ribonucleoprotein L	60	6.65	126	29	38	13	23
30	O60701	UDP-glucose 6-dehydrogenase	55	6.73	119	32	35	12	22
31	P06733	Alpha-enolase	47	6.99	97	43	64	13	47
32	P54868	Hydroxymethylglutaryl-CoA synthase	57	8.4	95	33	26	10	16
33	P14550	Alcohol dehydrogenase [NADP+]	36	6.35	94	31	39	9	29
34	Q16762	Thiosulfate sulfurtransferase	33	6.83	69	31	28	7	21
35	P05062	Fructose-bisphosphate aldolase B	39	8.06	80	37	37	9	26
36	P05062	Fructose-bisphosphate aldolase B	39	8.06	102	36	28	9	15
37	P22626	Heterogeneous nuclear ribonucleoproteins A2/B1	37	8.97	181	46	15	12	3
38	P22626	Heterogeneous nuclear ribonucleoproteins A2/B1	37	8.97	135	35	24	11	12
39	P21796	Voltage-dependent anion-selective channel protein 1	31	8.63	113	42	32	8	22
40	P17931[a)]	Galectin-3	26	8.6					
41	Q13268	Dehydrogenase/reductase SDR family member 2	27	8.9	88	36	41	9	31
42	Q06830	Peroxiredoxin 1	22	8.27	85	35	25	6	27

Spot No.	Swiss-Prot Acc. No.	Protein Name	M_r (kDa) th.	p*I* th.	MAS-COT Score	Seq. Cov. (%)	Nr. of Pept. Searched	Nr. of Pept. Matched	Nr. of Pept. Un-matched
43	P28062	Proteasome subunit beta type 8	30	7.63	74	30	38	7	30
44	P30086	Phosphatidylethanolamine-binding protein	21	7.43	117	59	40	8	30
45	P00915	Carbonic anhydrase 1	29	6.63	103	61	41	10	28
46	P18669	Phosphoglycerate mutase	29	6.75	70	37	28	7	21
47	P60174	Triosephosphate isomerase	27	6.51	230	81	37	13	20
48	P02768[b)]	Serum albumin	69	5.92	160	19	12	11	0
49-1	P30039	MAWD binding protein	33	5.64	83	32	31	6	23
49-2	P25786	Proteasome subunit alpha type 1	30	6.15	62	24	31	6	25
50	P50225	Sulfotransferase 1A1	34	6.16	92	41	29	8	20
51	P78417	Glutathione transferase omega 1	28	6.24	82	37	26	8	18
52	P50053	Ketohexokinase	33	5.64	70	37	30	7	23
53	P30084	Enoyl-CoA hydratase	31	8.34	105	29	11	6	4
54	P04792	Heat-shock protein beta-1	23	5.98	78	49	50	9	41
55	P30048	Thioredoxin-dependent peroxide reductase	28	7.68	85	28	21	6	13
56	P02768[b)]	Serum albumin	69	5.92	132	15	13	10	2
57	Q13162	Peroxiredoxin 4	31	5.86	117	51	54	10	42
58	P07339	Cathepsin D precursor	45	6.1	83	26	24	7	15
59	P09525	Annexin A4	36	5.85	124	35	26	11	13
60	P50224	Monoamine-sulfating phenol sulfotransferase	34	5.68	102	40	25	9	15
61	P35232	Prohibitin	30	5.57	74	28	25	7	18
62	P35232	Prohibitin	30	5.57	87	38	40	8	31
63	P28070	Proteasome subunit beta type 4	29	5.72	78	39	23	7	16
64	P09211	Glutathione S-transferase P	23	5.44	91	48	13	6	7
65-1	P30085	UMP-CMP kinase	22	5.44	97	51	25	7	17
65-2	P32119	Peroxiredoxin 2	22	5.67	69	28	25	5	19
66	Q9NRV9	Heme-binding protein 1	21	5.71	77	41	25	6	18
67	P02753	Plasma retinol-binding protein	23	5.76	77	45	37	7	29
68	O75947	ATP synthase D chain	18	5.22	71	36	11	4	6
69	P52565	Rho GDP-dissociation inhibitor 1	23	5.03	78	25	8	4	3
70	P07858[c)]	Cathepsin B precursor	38	5.88	69	25	17	6	10
71	P28066	Proteasome subunit alpha type 5	26	4.74	77	39	29	6	22
72	P31947	Epithelial cell marker protein 1	28	4.68	89	33	38	8	28
73	P15374	Ubiquitin carboxyl-terminal hydrolase isozyme L3	26	4.84	74	28	11	5	5
74	P13693	Translationally-controlled tumor protein	20	4.84	82	45	23	6	16
75	P28065	Proteasome subunit beta type 9	23	4.93	76	44	20	6	14
76	P07237[d)]	Protein disulfide-isomerase	57	4.76	77	8	5	4	0
77	P07237[d)]	Protein disulfide-isomerase	57	4.76	77	10	5	5	0
78	P62158	Calmodulin	17	4.09	68	45	15	4	10
79	P60660	Myosin light polypeptide 6	17	4.56	74	44	24	5	18
80	P60660	Myosin light polypeptide 6	17	4.56	80	34	10	4	5
81	P00167	Cytochrome b5	15	4.88	68	58	11	4	7
82	Q9Y2B0	MIR-interacting saposin-like protein precursor	21	4.81	69	28	17	4	10
83	P50120	Retinol-binding protein II, cellular	16	5.27	84	38	4	4	0
84	P09382	Galectin-1	15	5.34	70	52	34	6	28
85	P02766	Transthyretin	16	5.52	82	48	12	4	7
86	P12104	Fatty acid-binding protein, intestinal	15	6.88	74	45	23	5	17
87	P07148	Fatty acid-binding protein, liver	14	6.6	95	74	24	6	16
88	P62937	Peptidyl-prolyl cis-trans isomerase A	18	7.82	82	50	28	8	19

Spot No.	Swiss-Prot Acc. No.	Protein Name	M_r (kDa) th.	p*I* th.	MAS-COT Score	Seq. Cov. (%)	Nr. of Pept. Searched	Nr. of Pept. Matched	Nr. of Pept. Un-matched
89	P62937	Peptidyl-prolyl cis-trans isomerase A	18	7.82	84	60	49	6	41
90	P23528	Cofilin-1	18	8.26	88	61	47	6	39
91	P07737	Profilin-1	15	8.47	73	44	15	5	10
92	P68871	Hemoglobin beta	16	6.81	215	94	18	11	6
93	P69905	Hemoglobin alpha	15	8.73	71	38	7	4	3
94	P61604	10 kDa heat shock protein	11	8.91	99	43	6	4	1

a) Significant identification in K Lenaerts, et al. *Febs J* 2005, 272:3350-64
b) N-terminus
c) Peptide tolerance set to 120 ppm
d) C-terminus
Seq. cov., Sequence coverage; Nr. of pept., number of peptides
