# Supplementary material for: Comparative proteomic analysis of cell lines and scrapings of the human intestinal epithelium
Source: BMC Genomics. 2007 Apr 3;8:91. doi: 10.1186/1471-2164-8-91 (PMC1852558; doi:10.1186/1471-2164-8-91)
Supplement: Additional file 2 — Spot intensities of identified proteins. Averaged spot intensities with standard deviations of identified protein spots which are displayed in Figure 3 and 4. [file 1471-2164-8-91-S2.pdf]

Additional file 2 - Averaged spot intensities (Av) with standard deviations (St Dev) of identified protein spots which are displayed in Figure 3 and 4.

Spot No.	Small Int.	Large Int.	Caco-2 5d	Caco-2 15d	HT-29	Hep G2	TE 671
	Av St Dev	Av St Dev	Av St Dev	Av St Dev	Av St Dev	Av St Dev	Av St Dev
**Cluster 1**							
92	14185 ± 4893	12648 ± 1898	0 ± 0	0 ± 0	0 ± 0	0 ± 0	0 ± 0
93	10763 ± 2748	7592 ± 3876	0 ± 0	0 ± 0	0 ± 0	0 ± 0	0 ± 0
21	1714 ± 721	1466 ± 482	0 ± 0	0 ± 0	0 ± 0	0 ± 0	0 ± 0
56	1487 ± 161	5392 ± 1571	0 ± 0	0 ± 0	0 ± 0	0 ± 0	0 ± 0
45	963 ± 483	1622 ± 344	0 ± 0	0 ± 0	0 ± 0	0 ± 0	0 ± 0
85	642 ± 207	1300 ± 208	0 ± 0	0 ± 0	0 ± 0	0 ± 0	0 ± 0
18	1148 ± 142	1082 ± 314	83 ± 61	101 ± 71	104 ± 102	193 ± 123	78 ± 16
43	961 ± 268	405 ± 137	0 ± 0	0 ± 0	0 ± 0	0 ± 0	0 ± 0
33	1082 ± 369	604 ± 229	133 ± 27	187 ± 97	279 ± 50	510 ± 46	166 ± 169
61	1477 ± 648	940 ± 253	370 ± 120	422 ± 89	267 ± 178	404 ± 166	206 ± 46
86	5902 ± 588	496 ± 130	0 ± 0	0 ± 0	0 ± 0	0 ± 0	0 ± 0
44	5293 ± 743	1556 ± 788	316 ± 191	509 ± 194	1188 ± 751	1982 ± 884	654 ± 406
87	12066 ± 229	2860 ± 201	921 ± 102	2413 ± 868	0 ± 0	5633 ± 1497	0 ± 0
65	4944 ± 448	2954 ± 1307	1682 ± 262	1638 ± 504	2760 ± 443	3438 ± 878	2185 ± 305
90	5118 ± 1229	3608 ± 954	2289 ± 504	2695 ± 286	3312 ± 614	3209 ± 255	2033 ± 640
**Cluster 2**							
83	6097 ± 1333	0 ± 0	0 ± 0	0 ± 0	0 ± 0	0 ± 0	0 ± 0
36	5531 ± 859	0 ± 0	0 ± 0	0 ± 0	0 ± 0	0 ± 0	0 ± 0
35	2387 ± 326	0 ± 0	0 ± 0	0 ± 0	0 ± 0	0 ± 0	0 ± 0
52	1649 ± 300	0 ± 0	157 ± 54	95 ± 9	243 ± 76	149 ± 30	183 ± 35
75	1174 ± 219	230 ± 71	0 ± 0	0 ± 0	155 ± 170	0 ± 0	0 ± 0
81	827 ± 151	131 ± 136	60 ± 103	0 ± 0	113 ± 196	502 ± 93	0 ± 0
51	1399 ± 369	0 ± 0	252 ± 108	293 ± 112	525 ± 46	0 ± 0	128 ± 76
11	830 ± 196	140 ± 98	199 ± 78	161 ± 38	192 ± 68	236 ± 35	98 ± 30
49	1980 ± 259	219 ± 45	422 ± 104	464 ± 162	877 ± 85	1202 ± 241	774 ± 217
**Cluster 3**							
48	0 ± 0	613 ± 74	0 ± 0	0 ± 0	0 ± 0	0 ± 0	0 ± 0
**Cluster 4**							
27	205 ± 31	103 ± 30	1345 ± 259	1247 ± 213	868 ± 278	1065 ± 102	1190 ± 199
82	321 ± 103	171 ± 72	573 ± 198	831 ± 151	678 ± 243	1131 ± 11	1075 ± 381
4	502 ± 151	269 ± 279	1620 ± 597	1152 ± 78	2745 ± 529	1969 ± 197	998 ± 142
29	232 ± 94	137 ± 74	1054 ± 274	836 ± 186	894 ± 34	712 ± 122	1678 ± 105
2	0 ± 0	0 ± 0	760 ± 198	1164 ± 504	1826 ± 428	1211 ± 491	1049 ± 462
74	402 ± 329	229 ± 104	1304 ± 523	950 ± 385	2764 ± 474	1388 ± 46	1800 ± 167
80	0 ± 0	0 ± 0	1158 ± 105	526 ± 62	430 ± 261	1533 ± 120	295 ± 57
19	447 ± 93	302 ± 227	1407 ± 91	996 ± 407	1356 ± 173	835 ± 58	1065 ± 283
25	712 ± 196	286 ± 142	1271 ± 147	1204 ± 135	1004 ± 346	1137 ± 387	908 ± 134
3	493 ± 42	173 ± 168	1165 ± 193	1123 ± 131	1536 ± 340	1379 ± 205	1034 ± 254
38	480 ± 194	360 ± 132	1514 ± 553	1421 ± 164	1489 ± 276	903 ± 165	2717 ± 186
7	348 ± 135	247 ± 200	1459 ± 310	960 ± 62	1439 ± 355	1584 ± 194	1078 ± 512
1	1786 ± 383	621 ± 103	3457 ± 1292	4240 ± 679	2631 ± 307	3363 ± 844	3247 ± 114
57	541 ± 146	839 ± 115	1892 ± 55	1873 ± 276	947 ± 165	2338 ± 771	1622 ± 144
62	1577 ± 401	941 ± 366	5110 ± 524	4457 ± 942	2496 ± 478	3001 ± 198	3371 ± 953
37	1023 ± 578	1142 ± 346	5187 ± 2040	4679 ± 1056	4542 ± 418	2561 ± 318	6834 ± 1076
94	1289 ± 118	762 ± 265	3771 ± 1071	4226 ± 1437	2843 ± 1079	7838 ± 1895	5482 ± 2619
9	2161 ± 391	1537 ± 413	9195 ± 792	7712 ± 659	4866 ± 126	9910 ± 2890	5024 ± 297
**Cluster 5**							
72	846 ± 65	441 ± 79	234 ± 148	186 ± 56	1534 ± 21	276 ± 82	0 ± 0
34	644 ± 32	677 ± 105	600 ± 260	759 ± 152	326 ± 177	411 ± 122	0 ± 0
60	3360 ± 216	305 ± 109	526 ± 152	748 ± 58	684 ± 202	636 ± 135	0 ± 0
59	3376 ± 337	699 ± 337	1007 ± 244	1539 ± 292	2050 ± 173	970 ± 215	217 ± 51

Spot No.	Small Int. Av St Dev	Large Int. Av St Dev	Caco-2 5d Av St Dev	Caco-2 15d Av St Dev	HT-29 Av St Dev	Hep G2 Av St Dev	TE 671 Av St Dev
79	2649 ± 653	1369 ± 675	1693 ± 411	1047 ± 290	1828 ± 519	791 ± 100	70 ± 46
17	3970 ± 1103	654 ± 312	3156 ± 389	2936 ± 386	3932 ± 1726	1699 ± 131	0 ± 0
78	6962 ± 1480	3410 ± 1747	5191 ± 308	6006 ± 259	4175 ± 722	4161 ± 2210	1602 ± 756
**Cluster 6**							
8	0 ± 0	0 ± 0	0 ± 0	0 ± 0	0 ± 0	0 ± 0	3856 ± 237
84	340 ± 142	1695 ± 594	0 ± 0	617 ± 575	0 ± 0	610 ± 90	3258 ± 356
54	664 ± 238	914 ± 500	95 ± 126	289 ± 100	1385 ± 146	326 ± 96	3724 ± 860
6	84 ± 100	147 ± 96	179 ± 40	109 ± 51	821 ± 98	182 ± 7	1397 ± 126
**Cluster 7**							
32	2027 ± 468	541 ± 13	1219 ± 185	1017 ± 345	678 ± 105	0 ± 0	0 ± 0
16	2242 ± 517	367 ± 164	342 ± 64	717 ± 273	483 ± 104	0 ± 0	0 ± 0
10	1742 ± 282	714 ± 239	411 ± 102	444 ± 201	278 ± 95	0 ± 0	0 ± 0
64	4465 ± 329	2849 ± 2007	4928 ± 1065	4361 ± 224	6448 ± 584	0 ± 0	886 ± 109
**Cluster 8**							
23	359 ± 70	127 ± 63	408 ± 114	363 ± 87	163 ± 25	212 ± 30	135 ± 70
50	829 ± 129	0 ± 0	481 ± 77	464 ± 10	0 ± 0	117 ± 17	0 ± 0
28	1324 ± 179	87 ± 27	471 ± 116	541 ± 217	125 ± 57	322 ± 128	112 ± 35
26	1288 ± 123	296 ± 149	1269 ± 167	1107 ± 58	191 ± 121	314 ± 64	383 ± 144
66	2682 ± 627	129 ± 48	792 ± 156	1015 ± 333	113 ± 32	321 ± 18	210 ± 36
**Cluster 9**							
22	100 ± 2	0 ± 0	822 ± 187	738 ± 105	195 ± 130	200 ± 81	141 ± 35
14	575 ± 160	738 ± 141	2436 ± 730	2308 ± 406	335 ± 9	519 ± 115	921 ± 252
70	273 ± 178	325 ± 196	3362 ± 337	2978 ± 746	437 ± 223	1319 ± 254	1320 ± 602
53	2352 ± 730	764 ± 467	4625 ± 583	5403 ± 568	2557 ± 360	1533 ± 310	1837 ± 221
39	2072 ± 607	1016 ± 612	4939 ± 1542	5210 ± 1386	1135 ± 257	1621 ± 605	1869 ± 182
20	1006 ± 236	683 ± 328	3685 ± 1380	3886 ± 1360	1674 ± 265	1751 ± 592	1857 ± 563
55	1553 ± 581	800 ± 379	3450 ± 477	3543 ± 682	1981 ± 59	2076 ± 364	2164 ± 181
5	1647 ± 756	1192 ± 368	3250 ± 332	3253 ± 330	1753 ± 419	2144 ± 774	1854 ± 682
40	2216 ± 1047	2842 ± 1153	5583 ± 1244	7011 ± 1078	652 ± 136	621 ± 136	676 ± 434
**Cluster 10**							
73	180 ± 41	0 ± 0	188 ± 98	132 ± 24	764 ± 126	312 ± 37	65 ± 61
71	993 ± 137	399 ± 97	284 ± 105	189 ± 53	1354 ± 182	730 ± 122	546 ± 175
69	1454 ± 113	1070 ± 265	254 ± 52	283 ± 32	1311 ± 111	1211 ± 252	474 ± 122
24	1684 ± 429	696 ± 229	312 ± 91	362 ± 141	1838 ± 358	988 ± 113	254 ± 159
63	1295 ± 52	431 ± 293	606 ± 138	442 ± 56	1418 ± 202	815 ± 172	1966 ± 216
91	5044 ± 2137	5052 ± 2879	1526 ± 853	1134 ± 127	5025 ± 2336	3472 ± 293	5665 ± 1125
42	2922 ± 1120	2045 ± 437	1967 ± 766	1795 ± 202	3655 ± 1502	3292 ± 187	3799 ± 1172
**Cluster 11**							
30	206 ± 5	218 ± 78	116 ± 15	52 ± 12	147 ± 35	392 ± 68	102 ± 25
67	238 ± 137	593 ± 400	323 ± 123	579 ± 295	0 ± 0	2081 ± 222	0 ± 0
77	1392 ± 446	293 ± 94	327 ± 301	398 ± 156	287 ± 413	1524 ± 419	362 ± 138
13	1333 ± 466	353 ± 16	745 ± 399	864 ± 160	160 ± 88	1540 ± 63	597 ± 367
76	3226 ± 671	477 ± 153	1885 ± 1432	1421 ± 355	204 ± 208	3674 ± 605	1832 ± 984
41	2853 ± 492	0 ± 0	782 ± 315	767 ± 136	1015 ± 110	5136 ± 1614	632 ± 372
58	1655 ± 300	1724 ± 949	1659 ± 599	2013 ± 400	547 ± 106	1907 ± 341	701 ± 186
**Cluster 12**							
15	1477 ± 511	750 ± 622	1491 ± 566	1638 ± 457	705 ± 233	645 ± 94	1456 ± 673
46	1583 ± 291	1118 ± 338	1401 ± 305	1001 ± 142	1374 ± 76	1723 ± 356	988 ± 527
47	2808 ± 651	1573 ± 461	1866 ± 547	2091 ± 79	2286 ± 354	2204 ± 581	1160 ± 510
68	2008 ± 269	882 ± 485	2336 ± 616	1991 ± 546	1422 ± 508	2288 ± 308	1816 ± 181
31	2654 ± 265	2416 ± 640	1486 ± 267	1936 ± 869	2770 ± 79	3813 ± 1061	1870 ± 1051
88	4147 ± 852	3016 ± 1132	2309 ± 1069	1850 ± 640	4607 ± 158	3484 ± 657	4699 ± 1613
89	5761 ± 973	4843 ± 1297	3906 ± 1507	3279 ± 551	6042 ± 1276	4988 ± 1067	5475 ± 854
12	5985 ± 1266	4567 ± 1899	6100 ± 696	4908 ± 1409	5302 ± 695	4459 ± 1675	7144 ± 1559
